# Current status of the surgery-first approach (part I): concepts and orthodontic protocols

**DOI:** 10.1186/s40902-019-0194-4

**Published:** 2019-03-06

**Authors:** Dong-Soon Choi, Umberto Garagiola, Seong-Gon Kim

**Affiliations:** 1 Department of Orthodontics, College of Dentistry, Gangneung-Wonju National University, Gangneung, 25457 Republic of Korea; 2 Biomedical, Surgical and Oral Sciences Department, Maxillofacial and Dental Unit, School of Dentistry, University of Milan, Milan, Italy; 3 Department of Oral and Maxillofacial Surgery, College of Dentistry, Gangneung-Wonju National University, Jukheon gil 7, Gangneung, 25457 Gangwondo Republic of Korea

**Keywords:** Regional acceleratory phenomenon, Orthognathic surgery, Surgery-first approach, Orthodontics

## Abstract

The “surgery-first” approach, defined as a team approach between surgeons and orthodontists for orthognathic surgery without preoperative orthodontic treatment, is aimed at dental decompensation. A brief historical background and indications for the surgery-first approach are reviewed. Considering the complicated mechanism of postoperative orthodontic treatment, the proper selection of patients is a vital component of successful surgery-first approach.

## Background

When orthognathic surgery was introduced, every surgery was a surgery-first approach or surgery after completing orthodontic treatment [1]. This type of treatment had many problems such as postoperative occlusal instability and relapse [2]. Postoperative unstable occlusion results in serious problems in masticatory function. Accordingly, a three-stage approach (preoperative orthodontics, surgery, and postoperative orthodontics) has been set up and is considered to be the standard protocol [3, 4].

Recently, a precise treatment plan is possible with the help of three-dimensional (3D) imaging and simulation [5]. The development of a skeletal anchorage system can accelerate the speed of orthodontic treatment [6]. The discovery of surgery-facilitated orthodontics expands the understanding of postoperative tooth movement [7]. In clinical study, the serum level of alkaline phosphatase and type I collagen, which may be markers for bone turnover, is increased until 3 to 4 months postoperatively [8]. This is called the regional acceleratory phenomenon (RAP). RAP shows peak activity in 1 to 2 months after surgery and lasts until 6 to 24 months postoperatively in case of periodontal flap surgery [9]. When patients receive two-jaw surgery, the tooth mobility is increased from 1 week and shows similar level to their preoperative level in 4 months postoperatively [8]. In addition, patients dislike a long preoperative orthodontic treatment period [10, 11]. In the conventional approach, 15 to 24 months are required for preoperative orthodontics and an additional 7 to 12 months for postoperative orthodontics [12]. The surgery-first approach can reduce overall treatment time significantly [13]. These factors encourage the resurrection of the surgery-first approach.

Until now, there have been many pros and cons for the surgery-first approach. These debates may be similar to the debates when the three-stage approach was introduced. The people who supported the three-stage approach said that surgery-first is a dangerous approach, and the indication for this approach is very narrow. As postoperative occlusion is unstable in the surgery-first approach, postoperative relapse might be severe compared with the conventional three-stage approach [13]. As the surgery-first approach allows intensive tooth movement after surgery, the mandibular position immediately postoperative should not be a guide for the evaluation of postoperative relapse. In the treatment plan, posterior bite open and unstable occlusion are intentional, and vertical relapse should be evaluated after excluding these intentional factors.

It is true that there are easy cases and difficult cases in orthognathic surgery, in general. When a surgeon encounters a difficult case, an accurate treatment plan and active communication with orthodontists are basic requirements for successful treatment [14]. Accordingly, these debates primarily originate from the treatment plan for difficult cases. In this series of reviews, we want to discuss the current status of the surgery-first approach and the development of technology to expand the indications for the surgery-first approach.

## Main text

### History

The surgery-first approach has been widely accepted, and the setup protocol varies. As the first orthognathic surgery was done without preoperative orthodontics [1], the history of the surgery-first method may be the same as the history of orthognathic surgery. However, the current concept of surgery first is very different from the previous orthognathic surgery without orthodontic treatment. The first orthognathic surgery procedure was performed by Simon Hullihen in 1848. He published a paper in American Journal of Dental Science named “Case of Elongation of the Underjaw and Distortion of the Face and Neck, Caused by a Burn, Successfully Treated” in 1849, which is known as world’s first published paper about orthognathic surgery [15, 16]. He performed the first mandibular sub-apical osteotomy surgery to correct a protrusive malposed alveolar segment of the mandible. This surgical approach corrected the prognathism, but the patient showed anteriorly an edge-to-edge occlusion. Since, many new techniques and procedures as the conventional three-stage model of orthognathic surgery were later introduced and are still effective today in most cases. Dingman reported cases receiving surgery before orthodontics in 1944, but there was no comment on the role of orthodontists in the preoperative treatment plan [17]. The current concept of surgery first is the team approach between surgeons and orthodontists [14]. Therefore, surgery first without preoperative orthodontic consultation is not a surgery-first approach.

In 1959, Skaggs [18] suggested that patients with minor dentition problems may receive surgery before orthodontic treatment. However, this was a conceptual suggestion and not a team approach from the start. Later, Behrman and Behrman [19] proposed a concept similar to RAP. However, this was also a conceptual suggestion. Brachvogel et al. [20] suggested the potential advantages of a surgery-first approach. Most articles have cited the paper of Nagasaka et al. [21] in 2009 as the first clinical application of the surgery-first approach. As this case report described the first systematic team approach between orthodontists and surgeons, many authors recognized Nagasaka’s work as the first [14].

Since Nagasaka’s publication [21], the surgery-first approach has improved rapidly and has also been abused at times. Some surgeons performed “surgery first” without orthodontic consultation, and patients were referred to any orthodontist (personal observations). As surgery was done without any consideration for postoperative orthodontic treatment, some patients showed serious complications functionally and esthetically [22]. These malpractices are painful for the patients and increase the overall treatment period [22].

### Indications and limitations

**Fig. 1:**
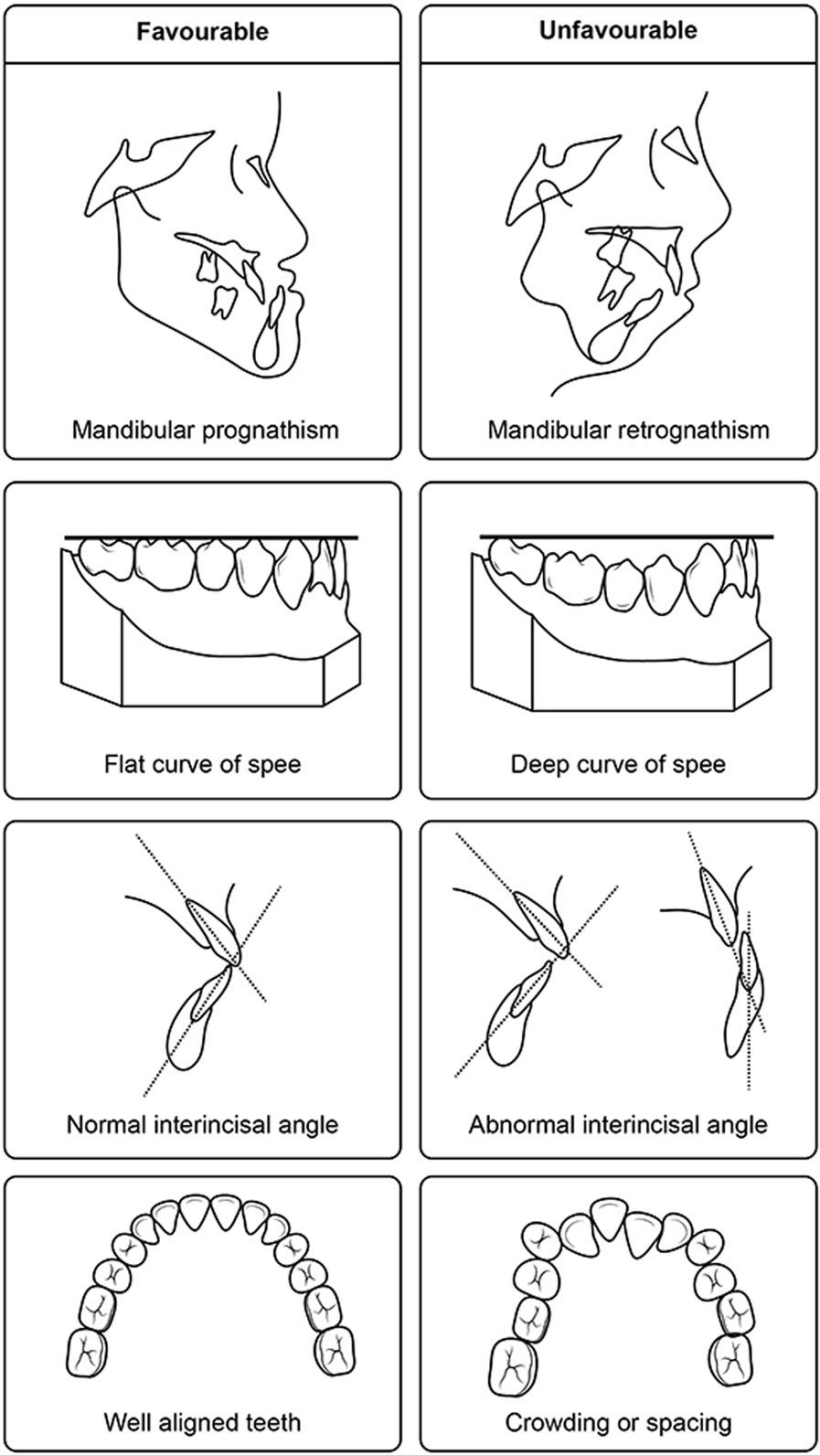
Favorable case and unfavorable case for the surgery-first approach. Some unfavorable cases may be considered for the surgery-first approach. However, much more sophisticated treatment plan is required for unfavorable cases

The surgery-first approach was developed to improve patient care. The first indication for the surgery-first approach should be patient demand [22, 23]. Patients, in general, do not like preoperative orthodontic treatment [10, 11]. The primary aim of preoperative orthodontics is decompensation and occlusal stability after surgery [12]. Accordingly, facial profile and preoperative occlusion are de-emphasized in preoperative orthodontic treatment [12, 24], making preoperative orthodontics even less attractive to patients. The surgery-first approach is basically a team approach between orthodontists and surgeons. Any surgery without a preoperative consultation between surgeons and orthodontists is inadvisable. Based on this consultation, the patients who do not require extensive preoperative orthodontics are indicated for the surgery-first approach [25]. The indications for the surgery-first patient are well summarized in a previous publication [26]. They are (1) minimal crowding in the anterior teeth, (2) favorable curve of Spee, and (3) normal range of angle between the basal bone to upper and lower incisors (Fig. 1).

Most patients with mandibular prognathism are indicated for the surgery-first approach [21, 27]. Because patients with class III prognathism with open bite usually have mild crowding and less dental compensation, they are good candidates for the surgery-first approach [28, 29]. Some patients with a narrow palatal arch need posterior impaction of the maxilla and posterior open bite [30]. With the help of a skeletal anchorage system, palatal arch expansion can be easily achieved [27]. If the arch discrepancy is attempted to be corrected by preoperative orthodontics, intercuspal interference makes the palatal expansion difficult. This movement can be done without cuspal interference in the case of posterior impaction by the surgery-first approach and a skeletal anchorage system. However, there is a concern about increasing occlusal vertical dimension [31]. When comparing the patients who have transverse discrepancy between the surgery-first approach and the conventional approach, there is no significant difference in tooth inclination between groups [30].

The patients with flat-to-mild curve of Spee will be considered for the surgery-first approach [26]. Patients with deep curve of Spee show a tendency to higher relapse at B-point [32]. In the course of flattening the curve of Spee, the mandible shows clockwise rotation because of posterior teeth extrusion [33]. In one study, intrusion of the anterior teeth was shown in the course of postoperative correction in the curve of Spee [32]. Dental crowding should be minimal [25, 34], and mild facial asymmetry is indicated for the surgery-first approach [25, 35].

If patients have favorable inter-incisal angle and alignment in the anterior teeth, the surgery-first approach is recommended [8]. In the viewpoint of postoperative occlusal stability, patients with a small overbite show better results than patients with a deep bite [31]. The patients who require less surgical movement have better stability after the surgery-first approach [31]. The recommended amount of mandibular setback for the surgery-first approach is less than 15 mm [31]. Patients with bimaxillary protrusion are also indicated for the surgery-first approach [25].

The limitation of the surgery-first approach is associated with occlusion at the time of surgery. The surgery-first approach cannot use the patient’s occlusion as a surgical movement [36]. Without the help of 3D virtual imaging and simulation surgery, complicated cases cannot be treated by the surgery-first approach [37]. As postoperative occlusion is generally unstable in the surgery-first approach, a surgical wafer should be maintained for guiding postoperative mandibular movement [37]. If there is a need for the application of surgical wire before surgery, any tooth movement should not occur preoperatively [38]. Patients with temporomandibular joint or the periodontal tissue problems may not be candidates for the surgery-first approach [14]. In case of mild temporomandibular disorder, the surgery-first approach with intraoral vertical ramus osteotomy may be considered [39]. The drawback of the surgery-first approach with intraoral vertical ramus osteotomy is 4 weeks of intermaxillary fixation [39]. As the peak activity of RAP is 1 to 2 months postoperatively [9], 4 weeks of intermaxillary fixation will delay the initiation of postoperative orthodontic treatment. The correction of mandibular retrognathism with deep bite, extraction case, and narrow palatal arch is not possible without preoperative orthodontic treatment [8, 26]. Most patients who are not recommended for the surgery-first approach require complicated postoperative orthodontic treatment.

However, the indication for the surgery-first approach has been widened with the help of new technology. Conventional treatment plans have been performed using two-dimensional (2D) dental models, such as a frontal and lateral cephalogram [40]. Cone-beam computed tomography (CBCT) allows a 3D treatment plan [28]. When CBCT is combined with 3D printing technology, a surgical wafer can be made by computer simulation [5]. These types of virtual treatment plans can avoid any error from face-bow transfer and dental model fabrication [5]. The virtual treatment plan may determine the osteotomy line and optimal position for rigid fixation with the help of a surgical guide [5, 41]. When a printed surgical wafer was compared to a conventional wafer, the printed wafer showed higher accuracy [42]. With the help of 3D virtual orthodontic simulation, orthodontists can accurately predict required tooth movement for the final occlusion [29]. To facilitate postoperative tooth movement, posterior bite open is common in the surgery-first approach and a skeletal anchorage system is mandatory to inhibit unwanted tooth extrusion [36]. Accordingly, the indication for the surgery-first approach has been continually widened.

### Orthodontic treatment after surgery

In the conventional orthodontics-first concept, pre-operative orthodontic treatment is provided to ensure the best possible position of dentition in the individual jaws prior to surgery, whereas the surgery-first approach provides the best possible normal jaw relations before the initiation of orthodontic treatment [21, 23]. Brachvogel et al. [20] mentioned that post-operative orthodontic treatment is similar to the dental arch alignment for class I malocclusion. In other words, if 3D skeletal discrepancies between the maxilla and the mandible are perfectly corrected with maxilla-mandibular surgery before orthodontic treatment, the postoperative orthodontic treatment is basically similar to the procedure for cases that have only dental malocclusions without any skeletal discrepancies. However, the post-operative orthodontic treatment in case of the surgery-first approach is inherently different from orthodontic treatment for dental class I malocclusion and post-operative orthodontic treatment of conventional orthodontics-first approach as well.

#### Goals of conventional orthodontic treatment and surgical-orthodontic treatment

The objectives of comprehensive orthodontic treatment are summarized as to achieve good alignment of dentition, to harmonize upper and lower dentition in three dimensions, and to improve occlusal interdigitation and dentofacial esthetics. In orthodontic camouflage treatment of skeletal malocclusion, the treatment objectives are compromised, and consequently, teeth positioning to the basal bone and facial esthetics may worsen. Therefore, the combination of orthodontic treatment for the dental malocclusion and surgical correction for the skeletal discrepancy would be the best choice in skeletal malocclusion. In this case, good interdisciplinary cooperation is essential to get the best outcome. Orthodontists and surgeons should be aware of each treatment objective, principles, and limits. In orthognathic surgery cases, the objectives of orthodontic treatment, extraction patterns, and types of mechanics used are frequently the reverse of those used in camouflage orthodontic treatment [43].

Understanding the objectives of orthodontic treatment in conventional orthognathic surgery, the orthodontics-first approach, is fundamental to understanding those in the new surgery-first approach. The conventional orthognathic surgery involves three steps: pre-operative orthodontic treatment, followed by orthognathic surgery and post-operative orthodontic treatment. The objective of pre-operative orthodontic treatment is to prepare the patient for surgery, and it is summarized as leveling and alignment of dental arches to eliminate any occlusal interference at surgery and removal of all dental compensations to maximize optimal surgical repositioning of the jaw. This pre-operative orthodontic preparation includes positioning of the incisors in ideal positions, establishment of good teeth inclination, and elimination of tooth-size discrepancies so as to permit class I canine and molar relationships [43]. To remove dental compensation in the sagittal plane, retracting the maxillary incisors and protracting the mandibular incisors are often required in skeletal class III malocclusion [44]. Inter-arch elastics, class II elastics in class III cases (and vice versa), temporary anchorage devices, such as orthodontic mini-screws, and strategic orthodontic extractions may be used for this dental decompensation. Pre-operative orthodontic treatment in the vertical plane focuses on vertical position of the incisors. This is essential so that the incisors will not interfere with repositioning the jaws in the desired position. In this concept, if the patient presents excessive facial height and deep curve of Spee, intrusion of the incisors must be accomplished pre-operatively [45]. Pre-operative orthodontic treatment should avoid compensatory teeth movements that may cause relapse tendencies after surgery; however, it should not always include pre-operative leveling of the curve of Spee, which can be done more efficiently during post-operative orthodontic treatment. In other words, since post-operative orthodontic treatment will be performed, some teeth movements may not be corrected prior to surgery. The objectives of conventional post-operative orthodontic treatment are summarized as follows: (1) stabilization of the occlusion after surgery, (2) additional leveling and alignment of the dental arch that is not completed during pre-operative orthodontic treatment, (3) coordinating both dental arches and sometimes inducing dental compensation depending on the postoperative skeletal relapse that may occur, and (4) settling the teeth into better interdigitation.

#### Goals of orthodontic treatment in orthognathic surgery using the surgery-first approach

The objectives of orthodontic treatment in the surgery-first approach are basically not different from those in the conventional orthodontics-first approach, in that the orthodontic treatment corrects mainly intra-arch dental problems and orthognathic surgery targets inter-arch problems originating from the skeletal discrepancy. Liao et al. [13] simply stated that the goals of post-operative orthodontics in surgery-first orthognathics are to decompensate the malocclusion, detail the occlusion, and ensure skeletal stability. In detail, however, there are quite a few differences in post-operative orthodontic treatment between the orthodontics-first approach and the surgery-first approach. Because orthodontic treatment is not performed pre-operatively in the surgery-first approach, there is almost unavoidable occlusal instability at surgery and the jaws may be repositioned to an undesired position due to occlusal interferences. Therefore, the main concerns are (1) how to manage the occlusal interferences during the stabilization period after surgery; (2) vertical and sagittal occlusal changes depending on autorotation of the mandible, which may occur by elimination of occlusal interferences; and (3) arch coordination and dental decompensation without triggering the jaws to return to their original position.

#### Pre-operative orthodontic protocols for the surgery-first approach

The literature about the surgery-first approach has been rapidly increasing over the last decade. Some of the articles were case-control studies [13, 31] and review articles [46, 47], but most of the articles were single-case reports [21, 27, 36] or case series (Table 1) [38, 48, 49]. The pre-operative orthodontic protocols varied between studies, or that information was unclear (Table 2). Some authors recommended 1 or 2 months of minimum preoperative orthodontic treatment in cases of severe occlusal prematurity [38], whereas many authors did not perform pre-operative orthodontic treatments in the surgery-first approach [13, 33]. The time of application of an orthodontic arch wire or surgical wire for intermaxillary fixation was 1 to 3 days [13, 48] or 2 to 3 weeks before surgery [49]. Hernandez-Alfaro et al. [48] placed four to eight mini-screws at the interdental area for intermaxillary fixation, another used brackets for the application of arch wires [13, 33], and others bonded the arch wires directly to the tooth surface [33, 38].

**Table 1:** Literature overview of orthognathic surgery using the surgery-first approach

Authors (year)	Study design	Sample size	Type of malocclusion	Presurgical orthodontic treatment	Surgical method	Fixation method	Total treatment time (months)
Baek et al. (2010)	Case series	11	Class III	1–2 months	LF + BSSO	NR	12.2 ± 3.6
Liao et al. (2010)	Case/control	20 SFA, 13 CA	Class III + open bite	No	LF + BSSO	Rigid fixation	SFA 11.4 ± 4.2 CA 17.1 ± 3.4
Wang et al. (2010)	Case/control	18 SFA, 18 CA	Class III	No	LF + BSSO	NR	NR
Ko et al. (2011)	Case/control	18 SFA, 35 CA	Class III	No	LF + BSSO	NR	SFA 17.8 ± 5 CA 15.8 ± 2.7
Hermandez-Alfaro et al. (2014)	Case series	45	Class II (19), class III (22), asymmetry (4)	No	LF + BSSO, LF, BSSO, others	NR	8.8
Kim et al. (2014)	Case/control	23 SFA, 38 CA	Class III	No	BSSO	Rigid fixation	SAF 15.4 CA 22.5
Kim et al. (2014)	Case series	37	Class III	No	LF + IVRO	IMF	14 ± 6
Choi et al. (2015)	Case/control	32 SFA, 24 CA	Class III	No	LF + BSSO	Rigid fixation	SFA 19.4 CA 22.3

*SFA* surgery-first approach, *CA* conventional approach, *LF* LeFort I osteotomy, *BSSO* bilateral sagittal split osteotomy, *IVRO* intraoral vertical ramus osteotomy, *NR* not reported

**Table 2:** Literature overview of post-operative orthodontic treatment in the surgery-first approach

Authors (year)	Bracket bonding before surgery	Arch wire placement before surgery	Use of skeletal anchorage for IMF	Duration of splint use	Initiation of post-operative orthodontic treatment after surgery	Use of inter-maxillary elastic for decompensation
Baek et al. (2010)	Using bracket or without bracket	Passive surgical wires	NR	4 weeks	4 weeks	Not clear (use of class III mechanics)
Liao et al. (2010)	1 month (022 slot)	1–3 days (016X022 NiTi)	NR	NR	Immediately after surgery	Class II elastics
Wang et al. (2010)	1–2 weeks (022 slot)	NR	NR	NR	NR	NR
Ko et al. (2011)	Before surgery	016 SS	NR	NR	Not clear (immediately after surgery)	NR
Hermandez-Alfaro et al. (2014)	1 week	1 day (soft wire)	4–8 mini-screws	2 weeks (only for maxillary segmental surgery)	2 weeks	Not clear (“Z” elastics for maxillary segmental surgery)
Kim et al. (2014)	Before surgery	Passive wires	NR	4–6 weeks (with intermaxillary elastics)	NR (4–6 weeks?)	NR
Kim et al. (2014)	NR	2–3 weeks (surgical wire)	NR	2 weeks with IMF + 2 weeks with class II elastics (for physiotherapy)	2 ± 1 months	NR
Choi et al. (2015)	Using bracket or without bracket	Passive surgical wires	NR	NR	NR	NR

*NR* not reported, *NiTi* nickel-titanium, *SS* stainless steel, *IMF* intermaxillary fixation

#### Stabilization after surgery and initiation of post-operative orthodontic treatment

The protocols of stabilization after surgery and initiation of post-operative orthodontic treatment also varied between studies or were not clearly described in the literatures. Kim et al. [49] evaluated the postoperative stability of the surgery-first approach using intraoral vertical ramus osteotomy (IVRO). The bony segments are not fixed during IVRO, and they maintained intermaxillary fixation for about 2 weeks. Then, class II guiding elastics were used for mandibular rehabilitation as active physiotherapy. Post-operative orthodontic treatment was initiated 2 months after surgery. Other studies on the surgery-first approach using bilateral sagittal split osteotomy (BSSO) reported mostly shorter stabilization time and earlier initiation of post-operative orthodontic treatment (ranging from immediately after surgery to 2 weeks after surgery) [13, 48]. However, Baek et al. [38] and Kim et al. [50] initiated the post-operative orthodontic treatment 4 to 6 weeks after surgery. There seems to be no consensus on the time of initiation of post-operative orthodontic treatment: immediately after, early, or delayed? Since the dental arches were not decompensated and harmonized prior to surgery, the occlusion is expected to be very unstable due to premature occlusal contacts. How long the surgical wafer should be maintained to cover the occlusal instability during bone healing period is a question to be answered. In the surgery-first approach using rigid fixation, is the long-term use of splint and delaying the post-operative orthodontic treatment reasonable to prevent early skeletal relapse? In contrast, Ko et al. [51] applied immediate post-operative leveling of the dentition to solve dental interference and arch compatibility. Changing heavy stabilization arch wires with light/resilient working wires immediately after surgery was suggested to shorten the post-operative orthodontic treatment time [45]. Other authors also emphasized that orthodontic treatment must start as soon as possible to take advantage of the regional acceleratory phenomenon after surgery [13, 48]. Orthognathic surgery might trigger 3 to 4 months of higher bone metabolism postoperatively, which might accelerate orthodontic tooth movement [8]. The protocols of stabilization and initiation of orthodontic treatment are still controversial with regard to post-operative stability and efficiency to shorten the time for post-operative orthodontic treatment.

#### Arch coordination and dental decompensation in post-operative orthodontics

It has been suggested that the advantage of the surgery-first approach is establishment of normal jaw relations before the initiation of orthodontic treatment. However, when the mandible sets back for class III malocclusion, it is often necessary to place the mandible in a somewhat clockwise-rotated position due to occlusal interferences. The counterclockwise rotation of the mandible would occur during post-operative orthodontic treatment, if the teeth that induced the occlusal interferences can be appropriately intruded. In conventional orthodontics, however, since extrusion of the teeth would occur more quickly and easily than intrusion of the teeth, using skeletal anchorage such as the orthodontic mini-screws may be essential especially in excessive facial height. The use of the skeletal anchorage for the arch coordination and dental decompensation was not clearly described in the literatures, whereas some authors used inter-maxillary elastics during post-operative orthodontic treatment [38, 43, 48]. Liao et al. [13] used class II elastics for incisor decompensation, and Hernandez-Alfaro et al. [48] used “Z” elastics for transversal control, whereas Baek el al. [38] probably used class III elastics to compensate for the postoperative relapse.

It may be impossible to standardize the protocols for post-operative orthodontic treatment, because the surgical-orthodontic treatment applied would vary considerably depending on the malocclusions and facial types of patients. However, the surgeons and orthodontists should be aware of the principles and limits of the surgery-first approach and predict positional change of the mandible by post-operative arch coordination and dental decompensation.

## Conclusion

The surgery-first approach has improved rapidly since its introduction. The indication for the surgery-first approach has widened with technical advancement. However, the limitations of this approach should be considered. Team approach between surgeons and orthodontists is a vital component for successful treatment.

## Abbreviations

2D: Two-dimensional
3D: Three-dimensional
BSSRO: Bilateral sagittal split osteotomy
CBCT: Cone-beam computed tomography
IVRO: Intraoral vertical ramus osteotomy
RAP: Regional acceleratory phenomenon

## References

[1] Poulton DR, Taylor RC, Ware WH (1963). Cephalometric X-ray evaluation of the vertical osteotomy correction of mandibular prognathism. Oral Surg Oral Med Oral Pathol.

[2] Bell WH, Creekmore TD (1973). Surgical-orthodontic correction of mandibular prognathism. Am J Orthod.

[3] Proffit WR, Miguel JA (1995). The duration and sequencing of surgical orthodontic treatment. Int J Adult Orthodon Orthognath Surg.

[4] Proffit WR, White RP (2011). Development of surgeon-orthodontist interaction in orthognathic surgery. Semin Orthod.

[5] Lee YC, Sohn HB, Kim SK, Bae OY, Lee JH (2015). A novel method for the management of proximal segment using computer assisted simulation surgery: correct condyle head positioning and better proximal segment placement. Maxillofac Plast Reconstr Surg.

[6] Huang CS, Chen YR (2015). Orthodontic principles and guidelines for the surgery-first approach to orthognathic surgery. Int J Oral Maxillofac Surg.

[7] Wilcko WM, Wilcko T, Bouquot JE (2001). Rapid orthodontics with alveolar reshaping: two case reports of decrowding. Int J Periodontics Restorative Dent.

[8] Liou EJW, Chen PS, Wang YC, Yu CC, Huang CS, Chen YR (2011). Surgery-first accelerated orthognathic surgery: postoperative rapid orthodontic tooth movement. J Oral Maxillofac Surg.

[9] Yaffe A, Fine N, Binderman I (1994). Regional accelerated phenomenon in the mandible following mucoperiosteal flap surgery. J Periodontol.

[10] Pelo S, Gasparini G, Garagiola U (2017). Surgery-first orthognathic approach vs traditional orthognathic approach: oral health-related quality of life assessed with 2 questionnaires. Am J Orthod Dentofac Orthop.

[11] Nurminen L, Pietilä T, Vinkka-Puhakka H (1999). Motivation for and satisfaction with orthodontic-surgical treatment: a retrospective study of 28 patients. Eur J Orthod.

[12] Luther F, Morris DO, Hart C (2003). Orthodontic preparation for orthognathic surgery: how long does it take and why? A retrospective study. Br J Oral Maxillofac Surg.

[13] Liao YF, Chiu YT, Huang CS, Ko EW, Chen YR (2010). Presurgical orthodontics versus no presurgical orthodontics: treatment outcome of surgical-orthodontic correction for skeletal class III open bite. Plast Reconstr Surg.

[14] Hernández-Alfaro F, Guijarro-Martínez R, Molina-Coral A (2011). “Surgery first” in bimaxillary orthognathic surgery. J Oral Maxillofac Surg.

[15] Aziz SR (2004). Simon P. Hullihen and the origin of orthognathic surgery. J Oral Maxillofac Surg.

[16] Goldwyn RM (1973). Simon P. Hullihen: pioneer oral and plastic surgeon. Plast Reconstr Surg.

[17] Dingman R (1944). Surgical correction of mandibular prognathism: an improved method. Am J Oral Surg.

[18] Skaggs JE (1959). Surgical correction of prognathism. Am J Orthod.

[19] Behrman SJ, Behrman DA (1988). Oral surgeons’ considerations in surgical orthodontic treatment. Dent Clin N Am.

[20] Brachvogel P, Berten JL, Hausamen JE (1991). Surgery before orthodontic treatment: a concept for timing the combined therapy of skeletal dysgnathias. Dtsc Zahn Mund Kieferheilkd Zentralbl.

[21] Nagasaka H, Sugawara J, Kawamura H, Nanda R (2009). “Surgery first” skeletal class III correction using the skeletal anchorage system. J Clin Orthod.

[22] Pelo S, Saponaro G, Patini R (2017). Risks in surgery-first orthognathic approach: complications of segmental osteotomies of the jaws. A systematic review Eur Rev Med Pharmacol Sci.

[23] Sarver D, Jacobson RS (2007). The aesthetic dentofacial analysis. Clin Plast Surg.

[24] O’Brien K, Wright J, Conboy F (2009). Prospective, multi-center study of the effectiveness of orthodontic/orthognathic surgery care in the United Kingdom. Am J Orthod Dentofac Orthop.

[25] Yu HB, Mao LX, Wang D, Fang B, Shen SG (2015). The surgery-first approach in orthognathic surgery: a retrospective study of 50 cases. Int J Oral Maxillofac Surg.

[26] Liou EJ, Chen PH, Wang YC (2011). Surgery-first accelerated orthognathic surgery: orthodontic guidelines and setup for model surgery. J Oral Maxillofac Surg.

[27] Villegas C, Uribe F, Sugawara J, Nanda R (2010). Expedited correction of significant dentofacial asymmetry using a “surgery first” approach. J Clin Orthod.

[28] Uribe F, Janakiraman N, Shafer D, Nanda R (2013). Three-dimensional cone-beam computed tomography-based virtual treatment planning and fabrication of a surgical splint for asymmetric patients: surgery first approach. Am J Orthod Dentofac Orthop.

[29] Hernández-Alfaro F, Guijarro-Martínez R (2014) On a definition of the appropriate timing for surgical intervention in orthognathic surgery. Int J Oral Maxillofac Surg 43: 846-855 10.1016/j.ijom.2014.02.007 24631424

[30] Wang YC, Ko EW, Huang CS, Chen YR, Takano-Yamamoto T (2010). Comparison of transverse dimensional changes in surgical skeletal Class III patients with and without presurgical orthodontics. J Oral Maxillofac Surg.

[31] Lee J, Kim YI, Hwang DS, Kim KB, Park SB (2014). Effect of occlusal vertical dimension changes on postsurgical skeletal changes in a surgery-first approach for skeletal Class III deformities. Am J Orthod Dentofac Orthop.

[32] Ko EW, Lin SC, Chen YR, Huang CS (2013). Skeletal and dental variables related to the stability of orthognathic surgery in skeletal Class III malocclusion with a surgery-first approach. J Oral Maxillofac Surg.

[33] Parker CD, Nanda RS, Currier GF (1995). Skeletal and dental changes associated with the treatment of deep bite malocclusion. Am J Orthod Dentofac Orthop.

[34] Choi JW, Lee JY, Yang SJ, Koh KS (2015). The reliability of a surgery-first orthognathic approach without presurgical orthodontic treatment for skeletal class III dentofacial deformity. Ann Plast Surg.

[35] Park HM, Lee YK, Choi JY, Baek SH (2014). Maxillary incisor inclination of skeletal Class III patients treated with extraction of the upper first premolars and two-jaw surgery: conventional orthognathic surgery vs surgery-first approach. Angle Orthod.

[36] Sugawara J, Aymach Z, Nagasaka DH (2010). “Surgery first” orthognathics to correct a skeletal Class II malocclusion with an impinging bite. J Clin Orthod.

[37] Hwang HS, Oh MH, Oh HK, Oh H (2017). Surgery-first approach in correcting skeletal Class III malocclusion with mandibular asymmetry. Am J Orthod Dentofac Orthop.

[38] Baek SH, Ahn HW, Kwon YH, Choi JY (2010). Surgery-first approach in skeletal Class III malocclusion treated with 2-jaw surgery: evaluation of surgical movement and postoperative orthodontic treatment. J Craniofac Surg.

[39] Park KR, Kim SY, Park HS, Jung YS (2013). Surgery-first approach on patients with temporomandibular joint disease by intraoral vertical ramus osteotomy. Oral Surg Oral Med Oral Pathol Oral Radiol.

[40] Schwartz HC (2011). Efficient surgical management of mandibular asymmetry. J Oral Maxillofac Surg.

[41] Hsu SS, Gateno J, Bell RB (2013). Accuracy of a computer-aided surgical simulation protocol for orthognathic surgery: a prospective multicenter study. J Oral Maxillofac Surg.

[42] Gateno J, Xia J, Teichgraeber JF, Rosen A, Hultgren B, Vadnais T (2003). The precision of computer-generated surgical splints. J Oral Maxillofac Surg.

[43] Jacobs JD, Sinclair PM (1983). Principles of orthodontic mechanics in orthognathic surgery cases. Am J Orthod.

[44] Grubb J, Evans C (2007). Orthodontic management of dentofacial skeletal deformities. Clin Plast Surg.

[45] Proffit WR, Fields HW, Sarver DM (2013). Combined surgical and orthodontic treatment, in contemporary orthodontics.

[46] Huang CS, Hsu SS, Chen YR (2014). Systematic review of the surgery-first approach in orthognathic surgery. Biom J.

[47] Peiro-Guijarro MA, Guijarro-Martinez R, Hernandez-Alfaro F (2016). Surgery first in orthognathic surgery: a systematic review of the literature. Am J Orthod Dentofac Orthop.

[48] Hernandez-Alfaro F, Guijarro-Martinez R, Peiro-Guijarro MA (2014). Surgery first in orthognathic surgery: what have we learned? A comprehensive workflow based on 45 consecutive cases. J Oral Maxillofac Surg.

[49] Kim JY, Jung HD, Kim SY, Park HS, Jung YS (2014). Postoperative stability for surgery-first approach using intraoral vertical ramus osteotomy: 12 month follow-up. Br J Oral Maxillofac Surg.

[50] Kim CS, Lee SC, Kyung HM, Park HS, Kwon TG (2014). Stability of mandibular setback surgery with and without presurgical orthodontics. J Oral Maxillofac Surg.

[51] Ko EW, Hsu SS, Hsieh HY, Wang YC, Huang CS, Chen YR (2011). Comparison of progressive cephalometric changes and postsurgical stability of skeletal Class III correction with and without presurgical orthodontic treatment. J Oral Maxillofac Surg.

